# The impact of workforce redesign policies on role boundaries in ‘generalist’ podiatry practice: expert views within the professional body

**DOI:** 10.1186/s13047-014-0052-7

**Published:** 2014-12-14

**Authors:** Samantha J Stressing, Alan M Borthwick

**Affiliations:** School of Medicine, Primary Medical Care, University of Southampton, Aldermoor Health Centre, Aldermoor Close, Southampton, SO16 5ST UK; Centre for Innovation and Leadership in Health Sciences, Faculty of Health Sciences, University of Southampton, Building 45, Highfield, Southampton, SO17 1BJ UK

**Keywords:** Workforce redesign, Role boundar, Podiatr, Generalis

## Abstract

**Background:**

Demographic changes and a predicted rise in the prevalence of chronic illness have led to a range of health policies in the UK (and elsewhere) focused on workforce flexibility and extended roles for the allied health professions. Whilst much academic attention has been paid to extended specialised roles for allied health professionals such as podiatrists, little work has addressed the likely impact of these policy changes on non-specialist, ‘generalist’ podiatry practice. This study aimed to explore expert professional views on the impact of role flexibility on generalist podiatry practice.

**Methods:**

Expert podiatry practitioners drawn from within the professional body, the Society of Chiropodists and Podiatrists/College of Podiatry were recruited to 3 focus groups and 4 individual semi structured interviews and the data subject to a thematic analysis.

**Results:**

Three key themes emerged, reflecting concerns about the future of generalist podiatry practice in the NHS, a perceived likelihood that generalist care will move inexorably towards private sector provision, and a growth in support worker grades undermining the position of generalist practice in the mainstream health division of labour. Up skilling generalist practitioners was viewed as the strongest defence against marginalisation.

**Conclusions:**

An emphasis on enhanced and specialised roles in podiatry by NHS commissioners and profession alike may threaten the sustainability of generalist podiatry provision in the state funded NHS. Non-specialist general podiatry may increasingly become the province of the private sector.

## Introduction

It is widely acknowledged that one of the key challenges for contemporary health service provision in Western democracies is an ageing population with a concomitant rise in the prevalence of chronic illnesses [1-4]. Demands on health services are set to rise as the demographic changes unfold. Policy level solutions have emphasised the need for workforce flexibility and an expansion in role boundaries for the allied health professions (AHPs), such as podiatry [1,5,6]. Much of the literature on workforce change in the health professions has focused on extended roles, such as prescribing or podiatric surgery, with little attention paid to the impact of these reforms on generalist practice [7-9]. This study aimed to address the issue of role flexibility and reform on non-specialist podiatry practice in the UK, often referred to as general podiatry or ‘core’ podiatry, through the eyes of expert podiatrists drawn from the ranks of its professional body [10,11].

## Background

Greater demands than ever before are being placed on the healthcare workforce as a result of demographic shifts evident since the post-World War 2 ‘baby boom’, and a growth in the prevalence of chronic illnesses, particularly relevant for the allied health professions [6,12,13]. By 2040 over 16 million people will have reached pensionable age in the UK, many of whom will increasingly become consumers of health services [14]. In addition, the healthcare workforce will continue to shrink, placing increasing strain on the capacity of the health services to cope with demand [15]. Financing a sustainable health service has become a significant challenge for providers, and redesign measures expanding the roles of allied health professionals have transformed the range of tasks and skills undertaken by podiatrists [2,7,8].

As professions respond to these shifts by developing increasingly specialised services, the ongoing place for non-specialists in contemporary healthcare is less clear. In the UK, policies such as ‘Meeting the Challenge’, were aimed at flexible working for AHPs, and stressed the growing specialisation of services as extended roles emerged [16]. Greater emphasis on skills and competencies for allied health professions implied a prioritization of extended, specialist roles, such as prescribing, alongside a growth in generic competencies for lower level work (such as assistant grade roles) [16,17].

As a result, questions now arise about the future role for generalist practitioners in the provision of foot care services in the NHS. Until now, generalist podiatry care has been provided by both the state-funded NHS system and the private sector, with most highly specialist roles being provided in hospitals or NHS primary care centres. Since 2010, Britain’s coalition government, in line with its neoliberal ideology, introduced further measures to enhance competition in the healthcare sector, with a view to encouraging a wider range of providers, including those in the private sector [18,19].

As a result, podiatry was targeted as one of eight services to be opened to the ‘Any Qualified Provider’ (AQP) scheme, enabling providers from the private sector to compete with existing providers in the NHS for contracts [18,20]. Importantly, the scheme was focused on the provision of non-specialist general podiatry [20]. Whilst elements of the scheme have subsequently been modified, it remains relevant as some services have been commissioned via this route, and the scheme reflects the prevalent policy agenda priorities. What, then, is likely to become of general podiatry care? Does such a move signal the gradual privatisation of general podiatry care, leaving the NHS to fund only specialist care of the ‘high risk’ foot?

This picture is complicated further by the inexact meanings assigned to the term ‘generalist’ and ‘specialist’ [2]. Podiatrists in general practice already delegate certain lower skill tasks to assistant grades, a common practice among health professions, and a growth in generic competency based practices may threaten the unique task domains of generalist providers [21].

More broadly, evidence suggests that current podiatry provision will be unable to meet the National Institute for Health and Care Excellence (NICE) short clinical guideline 87 in relation to diabetic foot treatment, which concerns both specialist and generalist care [22]. As diabetic foot disease is estimated to account for approximately 50% of the current NHS podiatry caseload, a projected gap of approximately 4,500 podiatrists is forecast [22]. However, concern has been expressed that generalist practitioners working in the private sector may lack some of the required skills, as the majority of people with diabetic foot complications tend to be treated in hospital or other NHS environments [22].

Currently, reduced NHS posts available to newly qualified graduates (as a result of fiscal constraints) inevitably means early career experience is likely to be gained via private sector care, where generalist roles predominate, with potential for an expanding skills gap, as opportunities to manage complex clinical cases may be reduced [23,24]. These factors draw into question the future for generalist roles in podiatry, and how they might best be delivered. This study aims to explore the views of experts within the profession, who are familiar with the health policy agenda and the trends in practice, on the future role and place of general practice in UK podiatry.

## Methods

A qualitative methodology incorporating focus group interview and face to face semi-structured interviews were adopted as appropriate to securing detailed, in-depth, informed comment and views from key actor experts within the professional body (The Society of Chiropodists & Podiatrists/College of Podiatry). Accessibility, feasibility and appropriateness were central considerations in selecting the methodology [25,26].

### Participant recruitment

The study was granted ethical approval through the Faculty of Health Sciences Ethics Committee at the University of Southampton. Podiatrists occupying senior roles in the professional body were recruited from the College Faculties of Management, Podiatric Medicine and General Practice, and the Society’s Private Practice Committee and Employment Relations Committee. Informed consent was received from each participant by the researcher prior to any research activity. Intensity sampling was appropriate to securing “information rich” cases, alongside snowball sampling [25].

### Data collection

Data were collected via semi-structured interviews and focus group interviews conducted at the offices of the professional body in central London. Three focus groups interviews were conducted, each with five to ten participants depending on respondent availability. Four face-to-face or telephone semi-structured interviews were conducted. Each was audio recorded and accompanied by a reflective diary. Transcription and data cleaning were completed prior to analysis by the researcher and an experienced departmental administrator using a transcription kit.

### Data analysis

Data were analysed with the aid of NVivo10 software, using a thematic approach [25]. Respondent validation of developing themes was used to enhance findings [25,26].

## Results

Sixty-one podiatrists were invited via the three selected groups from the SCP. Thirty-five expressions of interest were received but due to time constraints and other commitments, twelve of these individuals were unable to participate. Therefore, twenty-three podiatrists participated in three focus groups. Four participants took part in both a focus group interview and a face to face interview, making it possible to obtain greater in-depth information from these key informants.

The researcher originally intended to hold two focus groups but snowballing allowed a further focus group to be held following an invitation from the Employment Relations Committee. This was the most feasible recruitment method for the target audience, as the focus groups were held on dates coinciding with Faculty meetings.

Participants came from a range of backgrounds, including the NHS and private sector practice, in generic and specialist roles, NHS management, academia and education.

Three themes emerged from data analysis:

1. The Impact of Change
2. Concerns about Future Provision
3. Meeting the Challenge

### The Impact of Change

Participants attributed increased service demand and financial pressures as stimulating the development of a political agenda which encouraged generalist podiatry to be provided privately.

*“…I think [AQP] has demonised our profession by centralising core podiatry or generalist podiatry and moving it outside of the NHS…the specialist aspect of podiatry has been attached to all the professional areas, like diabetes, MSK and that the generalist podiatrist has been sort of almost marginalised…So therefore it is being seen as a private career.”* Participant 1018.

The participants perceived an increased uncertainty in clinical role boundaries, allowing the profession to become segregated into specialisms and across the devolved nations.

*“We…are actually creating more silos instead of becoming a unified profession…the podiatric surgeons, they wanted to be [on] their own and then…I can see that the diabetic specialists will maybe [will too]…then you will have the MSK set saying – well – if it's good enough for those two, we should really have ours – and I think that's where you kind of then come back to this – you know – it's that generalist – it's that private practitioner, where do they actually sit?…”* Participant 1057.

Changes in role boundaries were reported as having a negative impact on the recruitment and retention of staff.

### Concerns about Future Provision

Participants believed that removing the general podiatrist role could negatively impact on other groups, including specialists. The chief danger reportedly related to workload increases and reduced time for specialist work.

*“…I think it’s high end, high risk, the co-morbidity condition management stuff that is going to come towards the podiatrists and they either specialise or perish I think, ultimately…that's where the profession is going, but it comes at a cost because that high-end, high risk is very intense and you can't deliver the throughput…I think people have been asked to deliver a throughput and an intensity that's not sustainable in the long term.”* Participant 1059.

The participants demonstrated concern that the private sector may become flooded with clinicians no longer able to work in the NHS, reducing the workload for private practitioners already in the field and could allow private companies the opportunity to commandeer the market.

*“…the generalist is being forced out of the NHS, and if the NHS do not deliver generalist podiatry then it will be delivered by the private sector but not just by private practitioners but by other private healthcare companies…the impact on their lives, their terms and conditions, are major because these firms aren’t good employers and so part of the package of workforce redesign, forcing generalism out of the NHS, forcing podiatrists to work for companies like [private healthcare company] will have a major impact on them and the quality of the actual services that they deliver.”* Participant 1034.

It was perceived that service users have found their access to podiatry greatly reduced and inconsistent across the country.

*“…Depending in some places in England you have to be diabetic or have vascular disease or something high risk before you are allowed in, which is not equity of access at all because geographical differences are a bit unfair. But also it means that some people just because they have got a clinical condition can get podiatry treatment, regardless of the fact that they may not have that much wrong with their feet…”* Participant 1023.

It was felt that this inconsistency had led to an increased risk on an already vulnerable patient group, due to more self-treatment and service provision by lower graded staff. However, it was thought there was increased potential for patients to influence services.

*“…there’s, I believe…an opportunity for the public to influence this agenda… when the public have a much stronger influence on what…services that they are demanding…then that can easily influence…the commissioning…and that may not be…a way…that managers would historically look at being the most effective on a population base.”* Participant 1032.

Participants reported that improved patient choice in the NHS would go hand in hand with improved standards of care.

### Meeting the Challenge

The participants highlighted that further consideration may need to be given to the future of the generalist podiatrist, their skill mix and the potential to introduce more specialist generalists to allow these individuals to feel like valued members of the profession. The introduction of a clear career progression and academic structure may help justify their position in a clinical setting.

*“So what we've got to do as a profession in our side of workforce planning is to start planning the generalist – as a specialist and tell them where the equipment lies… ultrasound screening and vascular levels, all sorts of things…we can have a generalist who can do the job magically…I think that's going to be a bigger workforce planning thing and – I hope that locally, health and well-being boards, the health officers locally – will pick up the shortages of specialists and they will go for specialist generalists.”* Participant 1019.

Foundation degrees could also help to train lower graded individuals in order to match the trend towards more assistant bands in the NHS and also allow for better regulation of these members of staff.

*“…I think that the profession ultimately, in the long-term, will – will potentially change and you may find that foundation degrees…take over some of the stuff that is currently the preserve of a mainstream degree – at some stage in the future and there is a big part of the population that can be served by that level of competence, in my view, that doesn't actually need a degree to do it.”* Participant 1042.

The key message from the participants of this study was to encourage the podiatry profession to embrace change and support improvement.

*“…the challenge for the profession I think is change or die, bluntly. Because if you don't change you die, that’s what happened to dinosaurs so I think we have to make sure as a profession we support change, we support improvement …we need to fund them to do the change so that once austerity measures if you like have slackened off a bit, which we hope they will eventually, they're in a fit position to blossom.”* Participant 1059

## Discussion

It is clear, both from this study and from the wider literature, that contemporary healthcare provision is subject to demographic and policy changes that impact on the roles of the professions. As a result, the professions must adapt in order to retain a valued position within the health division of labour, and to meet an identified need in the population it serves. Whilst much of the literature to date has focused on the evolution of specialist practice, little attention has been paid to the impact of these changes on generalist podiatry care. In the UK the picture is also coloured by sector variance, with much of the specialist work becoming prioritised for NHS provision, and generalist care increasingly open to private sector commissioning. At the same time, generic working and the expansion in competency based skills affords great scope and enhanced roles for assistant grade workers across a wide spectrum of healthcare provision, possibly at the expense of generalist practitioners, whose role in the health division of labour is arguably becoming increasingly tenuous. At work in the shaping of these roles and the trend towards change are three key factors: demographic change, healthcare policy and professional aspirations. Healthcare policies clearly seek to ensure a sustainable healthcare provision into the longer term, through neoliberal solutions which involve the principles of market competition and patient ‘choice’. Demographic pressures, most notably the predicted (and evident) rise in chronic illness across an ageing population increase demand and thus generate ongoing funding crises. Finally, professional aspirations aiming to enhance prestige through the acquisition of advanced roles and technical skills and a need to maintain demand for services have implications for the way practice is adapted.

It is evident from the data in this study that the profession’s own experts, drawn from the upper echelons of the professional body, share a concern that generalist care is more vulnerable to challenge than other areas of practice. In part this is likely to result from both policy trends and professional aspirations. Almost all professions hold their specialist practitioners in high regard, esteemed as high status professionals undertaking ‘virtuoso’ roles, in stark contrast to those in general practice, which attracts much less prestige [21,27,28]. At the same time, NHS managers acknowledge the demographic realities and the need to address ever increasing demands from patients with chronic disease, often requiring specialist skills. It is also possible that the advancement of speciality roles reflects the higher educational demands made of these positions (such as Masters of Doctoral degrees at ‘consultant’ level), and the greater likelihood that such practitioners might generate research evidence supporting the case for specialisation. Commissioners of such roles are more likely to be persuaded to do so when evidence exists to support them. Also recognised as important is the need to utilise and extend generic working, in order to provide affordable care for a greater number of patients needing lower level care. In sum each of these scenarios appears to erode the ongoing requirement for generalist podiatry care in the NHS, in turn suggesting that the private sector may benefit. Already, schemes like ‘Any Qualified Provider’ indicate the direction of change, creating a concern among the respondents in the study that general practice may ultimately be outsourced to the private sector, or even, finally, abandoned to it.

Nor is it clear how the profession is likely to act collectively to adapt to these challenges whilst attempting to retain its existing privileges and place within mainstream healthcare. Professions operating exclusively in the private sector are often viewed as marginalised and lacking the recognition of established professions central to state provision [29,30]. One suggestion for establishing professional control over practice has been the regulation of assistant grade workers in podiatry, which is a common professionalising strategy designed to exclude competition and maintain autonomy [31]. This would, it is surmised, serve to protect the role and scope of the generalist practitioner whilst continuing to enable extensions of role boundaries at the upper end, in line with policy initiatives and professional aspirations. It would not, however, be likely to deflect the policy agenda sufficiently to prevent a significant growth in assistant workers, nor their acquisition of relevant competencies, given the underpinning drivers for such change. Nor would potentially new generic roles within general practice, such as health promotion, necessarily be sufficient to protect against generic worker empowerment. Other skills, at a level beyond that reasonable for assistant level workers, may be the only solution, requiring an upskilling across general practice. Yet, if general practice is to be provided in future in the private sector, opportunities for the full utilisation of these new skills may be less evident than in the NHS, resulting in an underused resource.

The findings in this study were necessarily constrained by certain limitations. Although key actors were recruited to the study, the sample size was still relatively small, given the range of speciality areas included. Face-to-face interviews, usually preferred for key actor interviews, were mainly conducted by focus group interview, in order to best utilise availability of participants. Finally, most participants were recruited from England, giving less opportunity for issues relating to the devolved administrations to be considered.

## Conclusions

In this study, the professional experts expressed a consensus view that upskilling in general practice would constitute the strongest defence for general practice, whilst acknowledging the possibility of private sector primacy in the delivery of general care in future.

## Abbreviations

AHP: Allied health professional
AQP: Any Qualified Provider
ESP: Extended scope practitioner
FCA: Foot care assistant
GP: General practitioner
NHS: National health service
PIS: Participant information sheet
SCP: Society of Chiropodists and Podiatrists
